# A Clinical and Biochemical Analysis in the Differential Diagnosis of Idiopathic Normal Pressure Hydrocephalus

**DOI:** 10.3389/fneur.2015.00086

**Published:** 2015-04-23

**Authors:** Tommaso Schirinzi, Giulia Maria Sancesario, Cristiano Ialongo, Paola Imbriani, Graziella Madeo, Sofia Toniolo, Alessandro Martorana, Antonio Pisani

**Affiliations:** ^1^Department of Systems Medicine, University of Rome “Tor Vergata”, Rome, Italy; ^2^IRCCS Santa Lucia Foundation, Rome, Italy; ^3^Department of Experimental Medicine and Surgery, University of Rome “Tor Vergata”, Rome, Italy

**Keywords:** idiopathic normal pressure hydrocephalus, CSF biomarkers, progressive supranuclear palsy

## Abstract

**Introduction:**

Idiopathic normal pressure hydrocephalus (iNPH) can be misdiagnosed with other neurodegenerative diseases, especially in the early disease stages. Considering the opportunity of the shunt surgery, iNPH should be diagnosed with accuracy. Here, we evaluate the utility of CSF biomarkers and their relationship with clinical features in the diagnosis of iNPH.

**Methods:**

We performed a multivariate analysis of the CSF levels of Aβ42, t-tau, and p-tau collected from four groups of patients: 14 iNPH, 14 progressive supranuclear palsy (PSP), 14 Alzheimer’s disease (AD), 14 controls (CTL). Diagnostic accuracy of biomarkers was determined by the receiver operating characteristic curve analysis. Statistical correlation was calculated between each CSF biomarker and single clinical items of iNPH.

**Results:**

Aβ42 levels in iNPH were lower than controls, although not as low as in AD. Likewise, CSF t-tau and p-tau were lower in iNPH than in controls. Of interest, t-tau and p-tau were higher in AD than in controls and hence both t-tau and p-tau were significantly lower in iNPH than in AD. No differences were found between iNPH and PSP. CSF biomarkers levels did not correlate to clinical features of iNPH, whereas two significant correlations emerged within clinical parameters: cognitive impairment was related to gait difficulties, while ventricular enlargement correlated with continence disturbances.

**Conclusion:**

Measurement of CSF biomarker levels may be helpful in the differential diagnosis between iNPH and AD but not between iNPH and PSP. Both Aβ42 and tau levels appear unrelated to main clinical features of iNPH.

## Introduction

Idiopathic normal pressure hydrocephalus (iNPH) is a syndrome characterized by enlargement of ventricular size with normal intracranial pressures along with the classic triad of dementia, gait disturbances and urinary incontinence (1, 2). However, early clinical features of iNPH may be subtle and lead to misdiagnosis with either neurodegenerative disorders or cerebrovascular diseases (1, 3). Indeed, the frontal dysexecutive syndrome, the most commonly reported cognitive profile of iNPH (4), is frequently observed in progressive supranuclear palsy (PSP) (5). Likewise, the characteristics of gait impairment observed in iNPH may not be of univocal interpretation, at least to some extent, thereby representing a confounding factor in the differential diagnosis with parkinsonian syndromes (3, 4). Therefore, considering that iNPH symptoms can be alleviated by appropriate shunt surgery (1), an improved accuracy in the diagnosis of iNPH should be pursued.

Recent studies support the usefulness of a combination of various CSF biomarkers of neurodegeneration to increase diagnostic accuracy during early phases of neurodegenerative diseases and iNPH (4, 6, 7). In this study, we measured a panel of CSF biomarkers, including 42 amino-acid forms of amyloid-β (Aβ42), total tau protein (t-tau), and phosphorylated tau protein (p-tau), to find elements supporting the differential diagnosis between iNPH and other neurodegenerative diseases. In addition, we performed a correlation analysis between clinical and biochemical features of iNPH.

## Subjects and Methods

### Subjects and clinical assessment

We enrolled a total of 56 subjects receiving lumbar puncture (LP) for diagnostic purposes admitted to the Neurology Unit of Policlinico Tor Vergata, Rome – Italy between 2012 and 2014. Subjects were divided into four groups. iNPH (*n* = 14), diagnosed according to iNPH guideline criteria for possible iNPH (8). Although both clinical and MRI criteria are considered sufficient to diagnose a possible iNPH (8), all iNPH patients underwent the spinal tap-test as further supportive diagnostic procedure (1, 8, 9). We considered a positive test if, 3 h after CSF drainage (30 ml), the time needed to walk 10 m (10-m straight walking test) was reduced by >20% (9).

For Alzheimer’s disease (AD) (*n* = 14) and PSP (*n* = 14), diagnosis was made according to internationally established operational criteria (10, 11). AD and PSP patients had mini mental state evaluation (MMSE) <26. Controls (CTL, *n* = 14) were non-demented patients, without evidence of other neurodegenerative disorders, undergoing LP for suspected chronic polyneuropathy. Before the CSF tap-test, iNPH patients underwent a rigorous assessment: cognitive decline was established through a complete psychometric evaluation (MMSE, Ray words test, Raven test, Rey–Osterrieth Complex Figure Test, Stroop test, verbal fluency test); the MMSE score, adjusted by age and educational level, was used as cognitive decline index. Gait and continence were estimated with the respective ordinal rating domain of the new iNPH scale (12); gait score was assigned observing tandem gait and turning (12). Evan’s index (EI) (8) was calculated through CT or MRI brain scans.

### CSF sampling and analysis

Lumbar puncture was performed following standard procedures as described previously (13, 14). All subjects were punctured in the morning of the same day of the clinical evaluation, lying in lateral position with atraumatic needles. CSF was collected in polypropylene tubes using standard sterile techniques. Blood specimens were also obtained at the same time of LP. Immediately after collection, CSF samples were stored on ice, sent to the local laboratory, and processed within 1 h. The first CSF sample was used for chemical and microscopic analysis (CSF samples containing >4 cells/μl would be excluded). The second sample was used for the determination of biomarkers levels through commercially available sandwich enzyme-linked immunosorbent assays following standard procedures (13, 14). To improve diagnostic accuracy, the Aβ42/p-tau ratio was calculated for each group (15). All procedures were carried out with the appropriate understanding and written consent of the subjects.

### Statistical analysis

A multivariate analysis of variance (MANOVA) with simple contrast was used to test the hypothesis of significance of the iNPH status as a factor compared to AD, PSP, and CTL for Aβ42, t-tau, and p-tau levels. The model was corrected for the main covariates in this study (age, gender, total CSF proteins, BBB index). Sensitivity and specificity of each biomarker were determined by the receiver operating characteristic (ROC) curve analysis, calculating the area under the curve (AUC), and the cut-off points. The Spearman’s correlation was used to test the association between Aβ42, t-tau and p-tau levels, total CSF proteins, BBB index in iNPH patients with other variables representative of the main clinical features of iNPH (MMSE, EI, gait and continence scores, disease’s duration). Statistical analysis was performed with SPSS 20, except for the power analysis, performed with GPower3.1.3. Data are presented as means ± SD.

## Results

**Figure 1:**
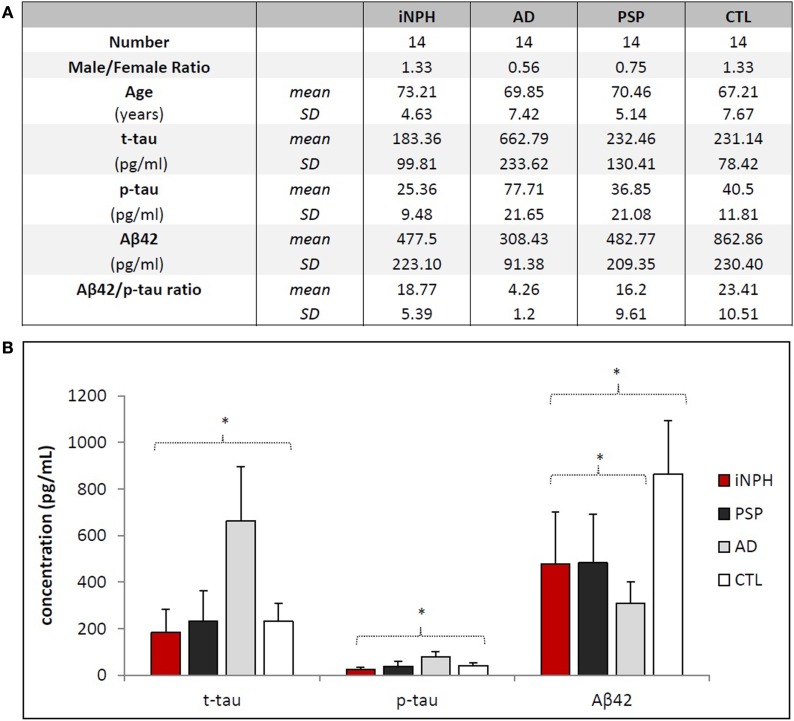
**Demographical and biochemical profiles of groups**. **(A)** No statistical differences exist regarding the distribution of age and gender among the groups. Aβ42/p-tau ratio was calculated considering ratio values <6.43 as AD, ≥6.43 as non-AD (14). Aβ42/p-tau ratio of AD group is significantly different (*p* < 0.01) from other groups. **(B)** Statistical differences of biomarkers concentrations among the groups. Asterisks indicate *p* < 0.05.

Demographic data and biomarker levels for each group are summarized in Figure 1. Clinical data of iNPH are reported in Table 1. Fourteen iNPH patients, 8 males and 6 females, had a mean age of 73.21 ± 4.63 years. Disease duration was 18.14 ± 8.75 months. MMSE score was 22.72 ± 4.95. Gait score was 4.21 ± 1.72. Continence score was 2.64 ± 1.34. EI was 0.33 ± 0.02.

**Table 1:** **Clinical data of iNPH**.

Patients	Age	Disease duration (months)	Gait score	MMSE	Continence score	Evan’s index
1	73	24	6	25.7	2	0.31
2	64	12	2	25.7	3	0.33
3	77	3	2	26.3	1	0.31
4	73	24	3	23.7	1	0.36
5	68	24	4	18	3	0.34
6	69	7	6	14.9	4	0.37
7	76	24	6	19.7	2	0.34
8	74	18	3	24.4	2	0.32
9	81	5	5	23.4	3	0.31
10	77	30	3	25	3	0.33
11	77	20	7	17.7	5	0.34
12	72	30	6	21.7	1	0.33
13	68	18	3	26.2	5	0.35
14	76	15	3	25.7	2	0.34
Mean	73.21	18.14	4.21	22.72	2.64	0.33
SD	4.63	8.75	1.72	4.95	1.34	0.02

Power analysis showed that a MANOVA model with 1 independent and 3 response variables, with 4 groups and a sample size of 56, reaches a power of 99% at a type I error level of 5% for a Cohen’s *f*^2^ of 0.2. The critical value of Spearman’s Rho coefficient for *N* = 14, a type I error level of 5%, and a two-tailed test was 0.538 (16). Statistical significance was assessed for a *p* < 0.05.

Mean levels of CSF biomarkers are significantly different among considered groups [*F*(3, 45) = 8.402, *p* < 0.01, Pillai’s trace = 1.047, partial η^2^ = 0.349] (Figure 1B).

CSF t-tau [*F*(3, 47) = 29.7, *p* < 0.01, partial η^2^ = 0.655] levels were slightly lower in iNPH than in CTL, although this result was far from statistical significance (*p* = 0.352). No significant difference was found between iNPH and PSP (*p* = 0.509). Conversely, t-tau was significantly lower in iNPH compared to AD (*p* < 0.01). To this regard, the ROC analysis provided an AUC of 0.99 (*p* < 0.01) and a cut-off value of t-tau <386 pg/ml (100% of sensitivity; 93.8% of specificity) in the discrimination of iNPH from AD (Table 2). Similar data were obtained with p-tau measurements [*F*(3, 47) = 20.2, *p* < 0.01, partial η^2^ = 0.563]. CSF p-tau was lower in iNPH than in CTL (*p* = 0.068) whereas no difference was measured with PSP (*p* = 0.114). Likewise, p-tau was lower in iNPH compared to AD (*p* < 0.01). ROC curve analysis provided an AUC of 0.99 (*p* < 0.01) and a cut-off value of p-tau <46 pg/ml (100% of sensitivity; 93.8% of specificity) in discriminating iNPH from AD (Table 2). Collectively, our data show that both t-tau and p-tau are higher in AD than in CTL and hence the CSF t-tau and p-tau are much lower in iNPH than in AD.

**Table 2:** **ROC curve analysis and cut-off values of CSF biomarkers levels in differential diagnosis between iNPH and AD**.

	t-tau	p-tau	Aβ42	Aβ42/t-tau
AUC	0.99	0.99	0.75	1
Cut-off value	<386 pg/ml	<46 pg/ml	>371 pg/ml	>6.43
Sensitivity (%)	100	100	73.30	100
Specificity (%)	93.8	93.8	81.30	93.80

CSF Aβ42 levels in iNPH group [*F*(3, 47) = 16.3, *p* < 0.01, partial η^2^ = 0.509] was significantly lower than in CTL (*p* < 0.01), though even lower levels were measured in AD (*p* = 0.01). No significant difference was found compared to PSP (*p* = 0.739). With respect to AD, ROC curve analysis provided an AUC of 0.75 (*p* = 0.02) and a cut-off value of Aβ42 >371 pg/ml (73.3% of sensitivity; 81.3% of specificity) to differentiate iNPH from AD (Table 2).

Our measurements were complemented by calculation of the mean values of Aβ42/p-tau ratio (15), summarized in Figure 1A. Notably, Aβ42/p-tau ratio values were fourfold lower in AD than in iNPH, suggesting that measurement of such ratio may significantly increase diagnostic accuracy in differentiating iNPH from AD. ROC analysis provided an AUC of 1.00 (*p* < 0.01). At the given cut-off value of 6.43 (15), we measured 100% of sensitivity and 93.8% of specificity in distinguishing between these diseases.

Correlation analysis showed no significant associations between t-tau, p-tau, Aβ42, total CSF proteins, and BBB index with iNPH clinical parameters. A negative correlation between MMSE and gait score (Rho −0.743, *p* < 0.01) was found, whereas a positive correlation was found between EI and continence score (Rho 0.594, *p* < 0.05). Values for each clinical parameter analyzed are reported in Table 1.

## Discussion

Idiopathic normal pressure hydrocephalus remains a controversial entity, believed to be determined by an imbalance in CSF turnover (1, 2, 7, 8). Notably, a significant proportion of the relatively few patients coming to autopsy have been shown to have co-existing neurodegenerative or vascular pathologies (3). Indeed, despite the potential different pathogeneses, some clinical features of iNPH, such as cognitive decline with selective impairment of the executive functions and attention (1, 4, 5), gait disturbances (3, 4), urinary urgency (17), and enlarging of ventricular system (18) may overlap with other neurodegenerative diseases such as AD and PSP. Considering the different therapeutic options, including CSF shunting for iNPH (1, 8), the search for better diagnostic investigations is mandatory.

It is well known that Aβ42 is reduced in CSF of patients with iNPH (4, 6, 7, 19). A number of different causes have been proposed to explain such reduction including a decreased production of amyloid-derived proteins, their impaired clearance from the extracellular fluid or their extracellular accumulation (7), or, finally, an aging-related phenomenon (20). Conversely, the literature reports no univocal changes of t-tau and p-tau levels in patients with iNPH (4, 6, 7, 19). In particular, the reduction of tau proteins has been referred to a phenomenon of dilution in the increased CSF volume of iNPH (4, 7).

In our study, we found that CSF levels of t-tau and p-tau are higher in AD than in CTL and hence t-tau and p-tau are much lower in iNPH than in AD, allowing to differentiate iNPH from AD. Furthermore, we observed that CSF Aβ42 levels of iNPH are lower than in CTL but not as low as it is in AD. Despite the small sample, our analysis provides plausible threshold values for CSF levels of t-tau, p-tau, and Aβ42/p-tau ratio, although larger samples are necessary to confirm these observations. Conversely, this panel of CSF biomarkers is not sufficient to discriminate iNPH from PSP.

Our findings also indicate that clinical parameters of iNPH, and in particular both cognitive decline and gait disturbances, despite the significant reduction of Aβ42 levels, are unrelated either to the β-amyloid pathology or to a tau-related degenerative process (7). However, it must be pointed out that these findings may have been affected by the relatively small number of subjects that warrants further investigations on larger sample groups.

Despite such limitation, our data suggest that the analysis of CSF biomarkers might be of support in the differential diagnosis of these different conditions, particularly in view of the distinct therapeutic options.

## Conflict of Interest Statement

The authors declare that the research was conducted in the absence of any commercial or financial relationships that could be construed as a potential conflict of interest.
